# Molecular Characterization and Functional Analysis of Annulate Lamellae Pore Complexes in Nuclear Transport in Mammalian Cells

**DOI:** 10.1371/journal.pone.0144508

**Published:** 2015-12-07

**Authors:** Sarita Raghunayakula, Divya Subramonian, Mary Dasso, Rita Kumar, Xiang-Dong Zhang

**Affiliations:** 1 Department of Biological Sciences, Wayne State University, Detroit, Michigan, United States of America; 2 Laboratory of Gene Regulation and Development, National Institute for Child Health and Human Development, NIH, Bethesda, Maryland, United States of America; 3 Departments of Emergency Medicine and Physiology, Wayne State University, Detroit, Michigan, United States of America; University of Toronto, CANADA

## Abstract

Annulate lamellae are cytoplasmic organelles containing stacked sheets of membranes embedded with pore complexes. These cytoplasmic pore complexes at annulate lamellae are morphologically similar to nuclear pore complexes at the nuclear envelope. Although annulate lamellae has been observed in nearly all types of cells, their biological functions are still largely unknown. Here we show that SUMO1-modification of the Ran GTPase-activating protein RanGAP1 not only target RanGAP1 to its known sites at nuclear pore complexes but also to annulate lamellae pore complexes through interactions with the Ran-binding protein RanBP2 and the SUMO-conjugating enzyme Ubc9 in mammalian cells. Furthermore, upregulation of annulate lamellae, which decreases the number of nuclear pore complexes and concurrently increases that of annulate lamellae pore complexes, causes a redistribution of nuclear transport receptors including importin α/β and the exportin CRM1 from nuclear pore complexes to annulate lamellae pore complexes and also reduces the rates of nuclear import and export. Moreover, our results reveal that importin α/β-mediated import complexes initially accumulate at annulate lamellae pore complexes upon the activation of nuclear import and subsequently disassociate for nuclear import through nuclear pore complexes in cells with upregulation of annulate lamellae. Lastly, CRM1-mediated export complexes are concentrated at both nuclear pore complexes and annulate lamellae pore complexes when the disassembly of these export complexes is inhibited by transient expression of a Ran GTPase mutant arrested in its GTP-bound form, suggesting that RanGAP1/RanBP2-activated RanGTP hydrolysis at these pore complexes is required for the dissociation of the export complexes. Hence, our findings provide a foundation for further investigation of how upregulation of annulate lamellae decreases the rates of nuclear transport and also for elucidation of the biological significance of the interaction between annulate lamellae pore complexes and nuclear transport complexes in mammalian cells.

## Introduction

**Fig 1:**
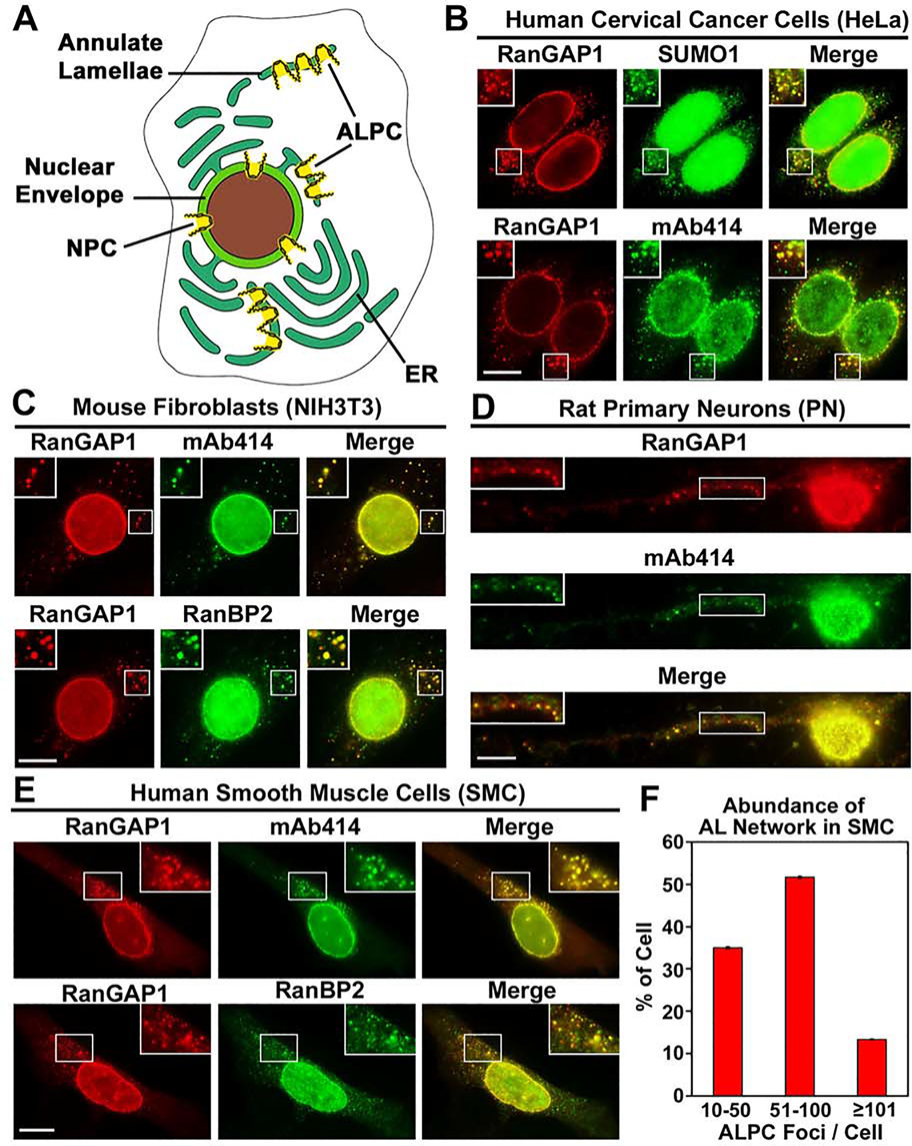
SUMO1-modified RanGAP1 localizes to both NPCs and ALPCs in a variety of mammalian cells. (A) The diagram shows that compared to NPCs in the nuclear envelope, ALPCs are embedded in the membrane cisternae of annulate lamellae that are often connected to the membrane network of ER. (B) Human cervical cancer cells (HeLa) were double-labeled with anti-RanGAP1 antibody and anti-SUMO1 mAb (21C7) or mAb414 for staining NPCs and ALPCs and then analyzed by immunofluorescence microscopy. (C) Mouse embryonic fibroblasts (NIH3T3) were double-stained with anti-RanGAP1 antibody and mAb414 or anti-RanBP2 mAb. (D) Rat primary cortical/hippocampal neurons (PN) were double-labeled with anti-RanGAP1 antibody and mAb414. (E) Human bronchial/tracheal smooth muscle cells (SMC) cells were double-stained with anti-RanGAP1 antibody and mAb414 or anti-RanBP2 mAb. Bar, 10 μm. The boxes at the top corner of each image show an enlarged version of inlets. (F) Annulate lamellae are highly abundant in SMC cells. 60 SMC cells were double-stained with anti-RanGAP1 antibody and mAb414. All the ALPC foci in each cell were counted under Olympus inverted IX81 fluorescence microscope using Z-stacks. The number of ALPC foci per cell was classified into three categories (10–50, 50–100 and ≥100), and the percentage of cells in each category was indicated. Each column represents the mean value ± SEM (*N* = 60) (ALPC foci/cell: 10–182; Average = 63).

Annulate lamellae are cytoplasmic organelles composed of membrane cisternae embedded with pore complexes [1]. These cytoplasmic pore complexes are structurally comparable to nuclear pore complexes (NPCs) at the nuclear envelope [1–5]. Like NPCs, annulate lamellae pore complexes (ALPCs) exhibit an eight-fold symmetrical structure and contain almost all the ~30 different NPC proteins (also called nucleoporins) except three nucleoporins including ELYS, POM121, and Tpr [6–10] (S1A Fig). Similar to the outer nuclear membrane of the nuclear envelope, annulate lamellae are often continuous with the membrane network of endoplasmic reticulum (ER) but not Golgi apparatus [1, 2, 4, 11–16] (Fig 1A). However, annulate lamellae lack the integral membrane proteins that are uniquely present in the inner nuclear membrane of the nuclear envelope for interaction with the nuclear lamina [17–21].

Annulate lamellae are often abundant in cells with high proliferative capacity, such as oocytes, embryonic cells and tumor cells [1, 4]. It has been considered that annulate lamellae may function as a storage compartment for excess nucleoporins to support the assembly of NPCs during rapid cell proliferation. However, studies of *Drosophila* early embryos show that the number of ALPCs does not decrease to compensate for the increasing number of NPCs [22]. Furthermore, annulate lamellae are also abundant in non-proliferating cells under permanent cell-cycle arrest, including murine neurons and cardiomyocytes [23, 24]. Intriguingly, levels of annulate lamellae can be upregulated by various treatments or conditions, such as antitubulin drugs, arginine deprivation, cell injury, reduced temperature, starvation, and irradiation [1]. Although annulate lamellae have been observed in almost all types of cells for over sixty years, their biological functions are still poorly understood [1, 25–28].

The translocation of most proteins into and out of the nucleus across NPCs is mediated by nuclear transport receptors called karyopherins including importins and exportins [29–31]. The best studied import pathway involves the importin α/β heterodimer, in which importin α acts as an adaptor and binds to import cargo with nuclear localization signal (NLS) in the cytoplasm. The importin α/β-cargo import complex passes across NPCs and is disassembled upon the bindings of importin β with RanGTP in the nucleus. The importin β-RanGTP complex exits the nucleus and is disassembled in the cytoplasm upon RanGTP hydrolysis, leading to the recycling of importin β for the next round of import. As the major exportin, CRM1 binds to export cargo with nuclear export signal (NES) in the presence of RanGTP in the nucleus, forming the CRM1-cargo-RanGTP complex, and releases the cargo upon RanGTP hydrolysis in the cytoplasm.

The Ran GTPase-activating protein RanGAP1 plays an essential role in nuclear transport by activating RanGTP hydrolysis at a physiologically significant rate [29, 30, 32, 33]. While unmodified RanGAP1 is predominantly cytoplasmic, modification by small ubiquitin-related modifier protein (SUMO) targets RanGAP1 to the cytoplasmic filaments of NPCs in vertebrate cells [34, 35]. At NPCs, SUMO1-modified RanGAP1 interacts with the Ran-binding protein RanBP2 (also called Nup358) and the SUMO-conjugating enzyme Ubc9, leading to the assembly of the RanBP2/RanGAP1*SUMO1/Ubc9 complexes [34–40]. As the major component of the cytoplasmic filaments of NPCs, RanBP2 contains multiple phenylalanine-glycine (FG) repeats for interacting with both importins and exportins, four RanGTP-binding domains, a zinc finger domain for specific interaction with CRM1, and an internal repeat (IR) or E3 ligase domain for interacting with SUMO1-modified RanGAP1 (RanGAP1*SUMO1) and Ubc9 [34–43] (S1 Fig).

It has been shown previously that NPCs function as the docking sites for the assembly of importin α/β-cargo import complexes through the interaction of importin β with FG-repeat containing nucleoporins such as RanBP2 during nuclear import [44–46]. Since RanGTP within CRM1-cargo-RanGTP and importin β-RanGTP complexes is not fully accessible by RanGAP1, the hydrolysis of RanGTP requires the coordination between RanGAP1 and its cofactor RanBP2 at the NPC or RanBP1 in the cytosol [29]. Hence, the RanBP2/RanGAP1*SUMO1/Ubc9 complex at the NPC provides a highly efficient platform for disassembling these RanGTP-containing transport complexes and therefore regulates both nuclear import and export [44, 45, 47, 48].

In this study, we show that both RanGAP1 SUMOylation and its interaction with RanBP2 are required for targeting RanGAP1 to annulate lamellae pore complexes by forming the RanBP2/RanGAP1*SUMO1/Ubc9 complexes in various types of mammalian cells. Furthermore, upregulation of annulate lamellae is accompanied with a redistribution of pore complexes and nuclear transport receptors including importin α/β and CRM1 from the nuclear envelope to annulate lamellae and also reduces the rates of both nuclear import and export. Lastly, our findings suggest a potential role of annulate lamellae pore complexes in nuclear transport through interaction with importin α/β-mediated import complexes and CRM1-mediated export complexes in mammalian cells.

## Materials and Methods

### Antibodies

Antibodies used in this study were obtained from the following sources: anti-RanGAP1 (19C7) and anti-SUMO1 (21C7) mouse monoclonal antibodies (mAbs) [35], Dr. Michael Matunis (Johns Hopkins, Baltimore, MD); anti-RanGAP1 rabbit polyclonal antibody (pAb) [49], Dr. Mary Dasso (NIH, Bethesda, MD); anti-POM121 rabbit pAb (EMD Millipore); anti-tubulin mouse mAb (Sigma); anti-Myc (9E10) mAb (Santa Cruz); anti-lamin B goat pAb (Santa Cruz); anti-nucleophosmin rabbit pAb (Santa Cruz); anti-RanBP2 mouse mAb (Santa Cruz); anti-RanBP2 rabbit pAb (Abcam); mAb414 mouse mAb (Covance); anti-Myc rabbit pAb (Cell Signaling); anti-calreticulin rabbit pAb (Calbiochem); anti-ELYS mouse mAb (Bio Matrix Research); anti-ELYS rabbit pAb (Bethyl Laboratories); anti-importin α mouse mAb (BD Biosciences); anti-importin β mouse mAb (BD Biosciences); anti-CRM1 mouse mAbs (BD Biosciences; Santa Cruz); anti-FLAG mouse mAb (M2) (Sigma); anti-FLAG rabbit pAb (Sigma); anti-Ubc9 rabbit pAb (Abcam); anti-Ran mouse mAb (BD Biosciences).

### Plasmids and siRNAs

The pAlter-Max plasmids encoding Myc-tagged RanGAP1 wild-type (WT) and K526R mutant were provided by Dr. Michael Matunis [50]. The pQE plasmids encoding His_6_-tagged human Ran WT and RanQ69L mutant were provided by Dr. Bryce Paschal (University of Virginia, Charlottesville, VA). The open reading frames of Ran WT and RanQ69L mutant were PCR amplified using the pQE plasmids as templates and subcloned into pFLAG-CMV2 vector for mammalian expression. The pXRGG and pXM10 plasmids were obtained from Dr. John Hanover (NIH, Bethesda, MD) for mammalian expression of Rev-GR-GFP shuttling cargos, in which the HIV-1 Rev protein contains NES WT (pXRGG) or mutant (pXM10) and the glucocorticoid receptor (GR) contains steroid-induced NLS [51]. The siRNAs specific to RanBP2 (CACAGAC AAAGCCGUUGAA) [45] and ELYS (AAUAUCUACAUAAUUGCUCUU) [52] and control non-targeting siRNAs (UUCUCCGAACG UGUCACGU) were purchased from Dharmacon.

### Cell culture, transfection and treatment

Five cancer cells, including HeLa (human cervical cancer cells) [53], HeLa stable cells expressing YFP-SUMO1 [54], BRL (Buffalo rat liver cells) [35], 293T (human embryonic kidney cells transformed with SV40 large T antigen) [38], and U2OS (human bone osteosarcoma cells) (a gift from Dr. Michael Matunis) [55], and two normal/non-tumorigenic fibroblasts, including GM03652 (human fibroblasts) [56] and NIH3T3 (mouse embryonic fibroblasts) (ATCC) were cultured in DMEM (Hyclone) supplemented with 10% fetal bovine serum (FBS) and 1% Penicillin-Streptomycin. The human bronchial/tracheal smooth muscle cells (SMC) (#FC-0059; Lifeline Cell Technology) were purchased and cultured the SMC cells according to the manufacturer’s protocols.

Primary cultures of a mixture of cortical and hippocampal neurons, which are referred to as primary neurons (PN), were isolated from embryonic day 18 Sprague–Dawley rats that are sacrificed by decapitation using a procedure as described previously [57] with a modification. All experiments were approved and conformed to the Wayne State University Institutional Animal Care and Use Committee (IACUC) as well as International Guidelines on the ethical use of animals. The animal care program at Wayne State University (WSU) has a Public Health Service (PHS) Office of Laboratory Animal Welfare (OLAW) animal use Assurance (assurance number A3310-01) and is registered as a research facility with the US Department of Agriculture (USDA), registration number 34-R-014. WSU is fully accredited by the Association for Assessment and Accreditation of Laboratory Animal Care International (AAALAC). All efforts were made to minimize the number of animals used and their suffering. In brief, cerebral cortices and hippocampi were isolated in ice-cold dissection buffer and incubated in papain. The tissue was then gently triturated in ice-cold Hibernate E medium (Invitrogen). After the tissue settled, the supernatant was aspirated, and the cells were resuspended in Neurobasal Media with B27 supplement (Invitrogen). Cells were plated on poly-d-lysine-coated plates and kept at 37°C in a 5% CO2 incubator. After 4–6 days *in vitro*, half the media was replaced and cultures were fed every 3–4 days. All experiments performed with a mixture of PN were used at 10–15 days *in vitro*. These cultures were 91–95% neuronal, as estimated by immunocytochemical staining according to the manufacturer’s protocols with anti-MAP2 antibody (1:10,000; #ab5392; Abcam).

The plasmids and siRNAs were transfected into HeLa cells using Lipofectamine-Plus reagent and Oligofectamine (Invitrogen), respectively. Vinblastine treatment and arginine deprivation were used to induce annulate lamellae. Vinblastine sulfate (Sigma) was dissolved in dimethyl sulfoxide (DMSO). HeLa cells were incubated with DMSO as a negative control or vinblastine sulfate at a final concentration of 0.1 μg/ml for 36 h. For arginine deprivation, HeLa cells were grown in the DMEM medium without L-lysine and L-arginine (Invitrogen) and supplemented with 10% dialyzed FBS (Invitrogen) and L-lysine (Sigma) in the presence (control) or absence of L-arginine (Sigma). L-lysine or L-arginine was added to a final concentration of 0.26 mM.

### Cell fractionation and immunoprecipitation

The nuclear and cytosolic fractions were obtained using either 0.015% Digitonin (Sigma) [35] or 0.5% NP-40 (US Biological) [58] as described previously. During the procedure of cell fractionation, 10 mM of N-ethylmaleimide (NEM) (Sigma) was used to inhibit deSUMOylation [53]. Total cell lysates and nuclear and cytosolic fractions were analyzed by immunoblotting with antibodies specific to SUMO1, RanGAP1, tubulin, lamin B, nucleophosmin, RanBP2, mAb414 and POM121. SUMO1-conjugates were immunoprecipitated from the nuclear and cytosolic fractions of HeLa cells with mAbs specific to SUMO1 (21C7) and analyzed by immunoblotting with antibodies specific to RanGAP1 and SUMO1. The mouse ascites generated using SP2/0 myeloma cells were used as a negative control for immunoprecipitation.

### Immunofluorescence microscopy

HeLa, BRL, 293T, U2OS, GM03652, NIH3T3 and HeLa YFP-SUMO1 stable cells were grown on coverslips, fixed with 2% formaldehyde in phosphate buffered saline (PBS) for 30 min at room temperature (RT), permeabilized with ice-cold acetone for 5 min, incubated with primary antibodies at RT for 1 h, stained with Alexa Fluor 488- and/or 594-conjugated secondary antibodies (Invitrogen) at RT for 30 min, and incubated in the mounting solution containing 4′,6-diamidino-2 phenylindole (DAPI) at RT for 5 min. The primary rat cortical/hippocampal neurons (PN) were fixed with 4% paraformaldehyde (Electron Microscopy Sciences) in PBS for 20 min, permeabilized with 0.5% Triton X-100 in PBS for 20 min, and then double-labeled with rabbit anti-RanGAP1 antibodies and mouse mAb414. The SMC cells were fixed with 3.5% paraformaldehyde in PBS for 30 min, permeabilized with 0.2% Triton X-100 in PBS for 5 min, and then double-labeled with rabbit anti-RanGAP1 antibodies and mouse mAb414 or anti-RanBP2 mAbs. Fluorescent images were taken by Olympus inverted IX81 fluorescence microscope with U-Plan S-Apo 60×/1.35 NA or 100×/1.40 NA oil immersion objective at 20°C and acquired with MicroSuite acquisition software (Olympus).

### Nuclear import and export assays

To test if induction of annulate lamellae by ELYS RNAi affects the rates of nuclear transport, HeLa cells were transfected with either control or ELYS-specific siRNAs for 48 h and then transfected with the constructs encoding Rev-GR-GFP fusion proteins for 24 h. To analyze the effect of annulate lamellae induction on the rates of nuclear import, the transfected cells were treated with 0.25 μM of dexamethasone (Calbiochem) for the indicated time and analyzed by immunofluorescence microscopy using rabbit anti-RanGAP1 antibody. To analyze if induction of annulate lamellae affects the rates of nuclear export, the transfected cells were treated with 0.25 μM of dexamethasone for 3 h to induce a nuclear accumulation of Rev-GR-GFP, washed with PBS buffer twice to remove dexamethasone, incubated with fresh media for nuclear export, and analyzed by immunofluorescence microscopy. For statistical analysis, the Rev-GR-GFP signals in the nucleus, cytoplasm and whole cell (total) were measured using ImageJ software (NIH). About 60 cells for each time point were analyzed to calculate the mean nuclear to total signal ratio of Rev-GR-GFP for nuclear import as well as the mean cytoplasmic to total signal ratio for nuclear export. Each bar represents the mean value ± SEM (Student’s *t* test).

### Analyses of nuclear import and export complexes at ALPCs

To test if the importin α/β-mediated import complexes are assembled at ALPCs, HeLa cells were first transfected with siRNAs specific to ELYS for 48 h to upregulate annulate lamellae and then the pXRGG plasmids encoding Rev-GR-GFP for 22 h. The transfected cells were treated with 200 nM leptomycin B (LMB) (Enzo Life Sciences) for 2 h to inhibit CRM1-mediated export, incubated with 1 μM dexamethasone and 200 nM of LMB for 0, 2.5, 5, 7.5, 15, 30 min to induce nuclear import of Rev-GR-GFP, and analyzed by immunofluorescence microcopy using mAb414.

To test if RanGTP hydrolysis takes place at ALPCs, HeLa cells were transfected with the constructs encoding FLAG-tagged Ran WT or RanQ69L mutant, double-stained with anti-FLAG and anti-RanGAP1 antibodies or mAb414, and analyzed by immunofluorescence microscopy. To test if ALPCs serve as cytoplasmic sites for the disassembly of CRM1-mediated export complexes by activating RanGTP hydrolysis, cells were co-transfected with the constructs encoding Rev-GR-GFP and FLAG-tagged Ran WT or RanQ69L mutant, incubated with dexamethasone for 10 min to induce import, washed and incubated with fresh medium to initiate nuclear export, stained with anti-RanGAP1 or anti-FLAG antibodies, and analyzed by immunofluorescence microscopy. The transfected cells were also doubled stained with mouse anti-FLAG and rabbit anti-RanGAP1 antibodies, and incubated with Alexa Fluor 350-conjugated goat anti-mouse and Alexa Fluor 594-conjugated goat anti-rabbit secondary antibodies for immunofluorescence microscopy.

## Results

### The RanBP2/RanGAP1*SUMO1/Ubc9 complexes are present at ALPCs in a variety of mammalian cells

Previous studies have shown that RanGAP1 and RanBP2 co-localize at cytoplasmic foci in HeLa cells [40], whereas YFP-tagged SUMO1 are found at cytoplasmic dots in HeLa YFP-SUMO1 stable cells [54]. However, it remains unclear whether these cytoplasmic foci/dots definitely represent the cytoplasmic pore complexes of annulate lamellae and whether SUMO1-modified RanGAP1, RanBP2 and Ubc9 also form the RanBP2/RanGAP1*SUMO1/Ubc9 complexes at ALPCs in mammalian cells. To address this question, we first tested if endogenous SUMO1 is co-localized with RanGAP1 at the ALPCs in mammalian cells. We double stained HeLa cells with rabbit anti-RanGAP1 antibody [49] and mouse mAb414 or anti-SUMO1 mAb [35] followed by immunofluorescence microscopy. The mAb414 has been widely used to label both NPCs and ALPCs as it stains multiple FG-repeat nucleoporins (including Nup62, Nup153 and RanBP2) [7, 8, 12, 59, 60]. Previous studies using immunoelectron microscopy and immunofluorescence microscopy have demonstrated that the cytoplasmic pore complexes labeled by mAb414 are associated with membranes and therefore represent ALPCs in mammalian cells [12, 60]. We found that RanGAP1 was almost completely co-localized with mAb414 at both NPCs and ALPCs, suggesting that RanGAP1 can be used as a marker for both pore complexes (Fig 1B, bottom panel). Furthermore, we showed that endogenous SUMO1 co-localized with RanGAP1 at both NPCs and ALPCs (Fig 1B, top panel), suggesting the presence of endogenous SUMO1-modified RanGAP1 at both pore complexes in HeLa cells.

To exclude the possibility of non-specific staining of anti-SUMO1 mAb at ALPCs (Fig 1B), we stained the HeLa YFP-SUMO1 stable cells [54] with anti-RanGAP1 antibody or mAb414 followed by immunofluorescence microscopy. We found that YFP-SUMO1 co-localized with RanGAP1 or mAb414 staining at both NPCs and ALPCs (S2A Fig), suggesting the presence of YFP-SUMO1-modified RanGAP1 at both pore complexes. Consistent with this, our immunoblot analysis of HeLa YFP-SUMO1 stable cells showed that RanGAP1 was robustly modified by both endogenous SUMO1 and YFP-SUMO1 in the HeLa stable cells (S2B Fig).

To test if RanGAP1 is also present at ALPCs in other types of mammalian cells, we double stained mouse embryonic fibroblasts (NIH3T3), primary rat cortical/hippocampal non-proliferating neurons (PN), and human bronchial/tracheal smooth muscle cells (SMC) with anti-RanGAP1 antibody and mAb414 or anti-RanBP2 antibody for immunofluorescence analysis (Fig 1C–1E). We found that RanGAP1 localized at both NPCs and ALPCs labeled by mAb414 and anti-RanBP2 antibody in these cells, suggesting the presence of both RanGAP1 and RanBP2 at ALPCs in a variety of mammalian cells (Fig 1C–1E). Among all the different cells that we analyzed, SMC cells have the highest average number of ALPC foci (~63 foci/cell) with a range of 10–182 foci/cell, indicating that every single SMC cell contains at least 10 ALPC foci (Fig 1F). On the other hand, HeLa cells, for example, only comprise an average of ~12 foci/cell with a range of 0–50 foci/cell and with ~15% of the cells with no obvious ALPC foci. This result suggests that the SMC cells may represent a suitable model for studying ALPCs in mammalian cells.

**Fig 2:**
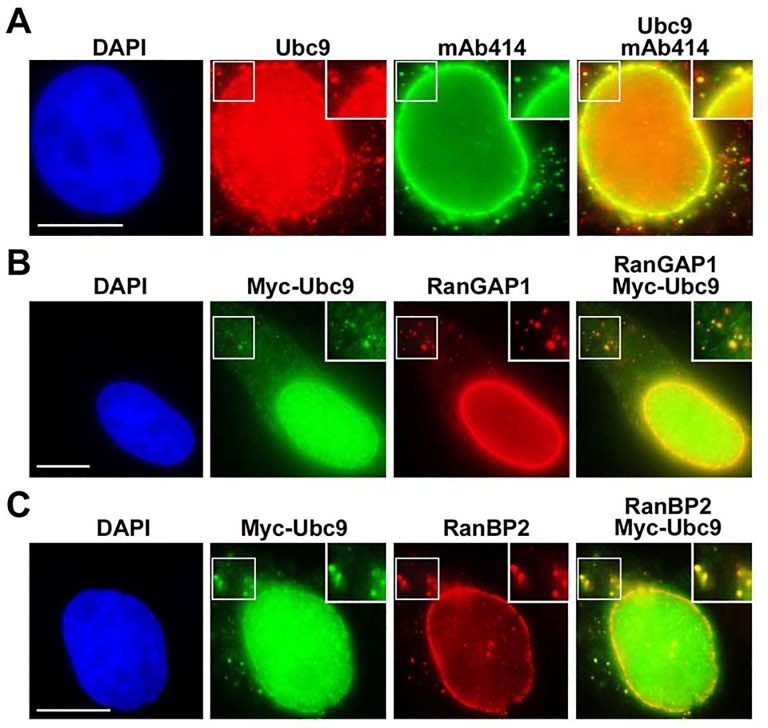
Ubc9 co-localizes with SUMO1-modified RanGAP1 and RanBP2 at both NPCs and ALPCs. (A) HeLa cells were analyzed by immunofluorescence microscopy using anti-Ubc9 antibody and mAb414. (B and C) HeLa cells were transfected with the construct encoding Myc-tagged Ubc9, double-stained with mouse anti-Myc mAb (9E10) and rabbit anti-RanGAP1 antibodies or with rabbit anti-Myc antibody and mouse anti-RanBP2 mAb, and then analyzed by immunofluorescence microscopy. Bar, 10 μm. The enlarged versions of inlets are shown at the top-right corner of each image.

The presence of endogenous SUMO1, RanGAP1, and RanBP2 at ALPCs prompted us to ask if Ubc9, a component of the RanBP2/RanGAP1*SUMO1/Ubc9 complex, is also present at ALPCs. Our immunofluorescence analysis showed that endogenous Ubc9 localized at both NPCs and ALPCs labeled by mAb414 (Fig 2A). To exclude a non-specific staining at ALPCs by anti-Ubc9 antibody, cells expressing Myc-tagged Ubc9 were double stained with mouse anti-Myc mAb and rabbit anti-RanGAP1 antibody or rabbit anti-Myc antibody and mouse anti-RanBP2 mAb for immunofluorescence microscopy (Fig 2B and 2C). We found that Myc-Ubc9 co-localized with RanGAP1 or RanBP2 at both NPCs and ALPCs. Altogether, our results suggest that the RanBP2/RanGAP1*SUMO1/Ubc9 complexes are also present at ALPCs in various types of mammalian cells.

### RanGAP1 localization to ALPCs depends on its SUMOylation and interaction with RanBP2

**Fig 3:**
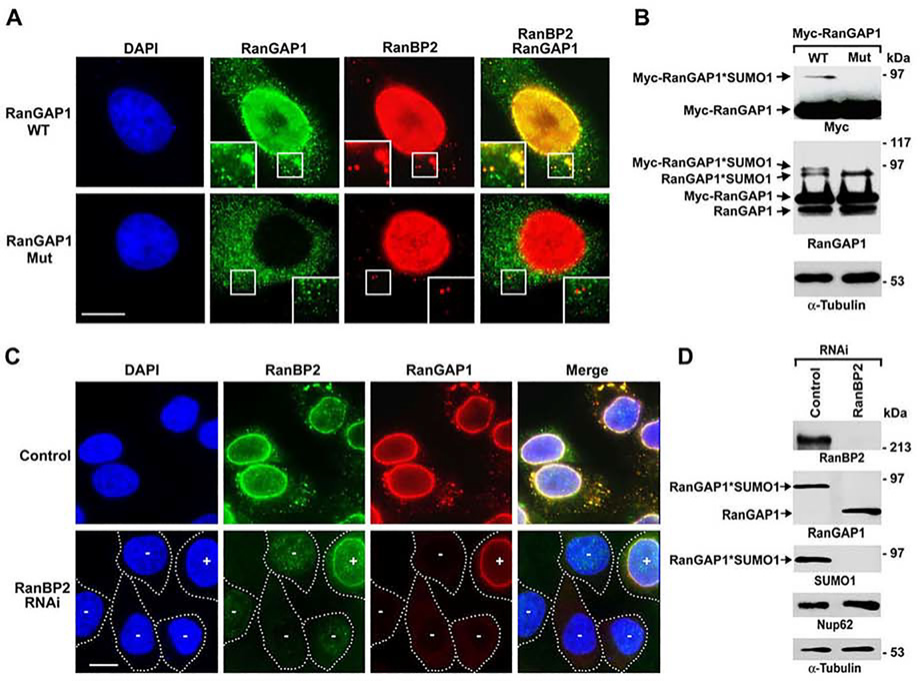
Both covalent SUMOylation and non-covalent interaction with RanBP2 are required for RanGAP1 localization to ALPCs. (A) HeLa cells were transfected with the constructs encoding Myc-tagged RanGAP1 wild-type (WT) or SUMOylation-deficient K526R mutant (Mut) and analyzed by immunofluorescence microscopy with antibodies specific to Myc and RanBP2. The boxes at the bottom corner of each image show the enlarged version of inlets. Bar, 10 μm. (B) The transfected cells were analyzed by immunoblotting with antibodies specific to RanGAP1, Myc and α-tubulin. (C) HeLa cells were transfected with control or RanBP2-specific siRNAs, double-stained with antibodies specific to RanBP2 and RanGAP1, and analyzed by immunofluorescence microscopy. In the lower panel, white dashed lines indicate the borders of RanBP2 RNAi cells, in which “-” indicates a significant knockdown of RanBP2 and “+” indicates that the signals of RanBP2 are comparable to those in control RNAi cells (upper panel). Bar, 10 μm. (D) The cells transfected with control or RanBP2-specific siRNAs were analyzed by immunoblotting with the indicated antibodies.

Based on mechanisms underlying RanGAP1 localization at NPCs [34–40, 50, 61], we hypothesized that both RanGAP1 SUMOylation and its interaction with RanBP2 are required for its localization at ALPCs. To test if SUMOylation of RanGAP1 is required for its localization to ALPCs, HeLa cells were transfected with the constructs encoding Myc-tagged RanGAP1 wild-type (WT) or its SUMOylation-deficient K526R mutant [50] and then analyzed by immunofluorescence microscopy using anti-Myc and anti-RanBP2 antibodies. We found that Myc-RanGAP1 WT but not its K526R mutant co-localized with RanBP2 at NPCs and ALPCs (Fig 3A). Furthermore, our immunoblot analysis confirmed that Myc-RanGAP1 WT but not its K526R mutant was SUMOylated (Fig 3B). We therefore concluded that SUMOylation of RanGAP1 is required for its localization at ALPCs.

To test if RanBP2 is required for RanGAP1 localization to ALPCs, HeLa cells were transfected with control non-targeting siRNAs or siRNAs specific to RanBP2 followed by immunofluorescence microscopy using anti-RanBP2 and anti-RanGAP1 antibodies (Fig 3C). Compared to the strong staining of RanBP2 and RanGAP1 at both pore complexes in control cells, RNAi-knockdown of RanBP2 abolished the localization of RanGAP1 at both NPCs and ALPCs (Fig 3C). SUMO1-modified RanGAP1 within the RanBP2/RanGAP1*SUMO1/Ubc9 complexes complex is known to be well protected from isopeptidase-mediated deSUMOylation (Zhu *et al*., 2009). Consistent with this, our immunoblot analysis showed that RNAi-depletion of RanBP2 caused a nearly complete loss of SUMO1-modified RanGAP1 along with an equivalent increase of unmodified RanGAP1 (Fig 3D). Hence, both RanGAP1 SUMOylation and its interaction with RanBP2 are required for its localization at ALPCs.

### The RanBP2/RanGAP1*SUMO1/Ubc9 complexes at ALPCs are distributed within ER network

**Fig 4:**
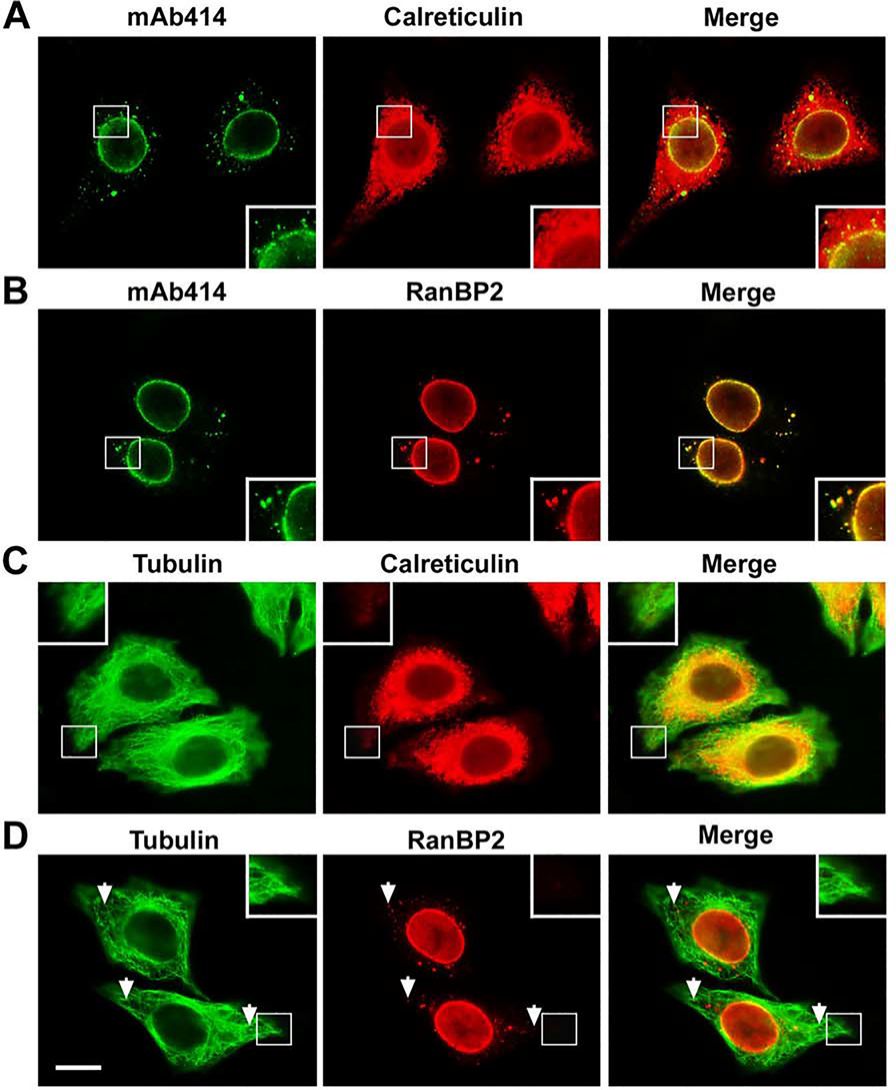
The ALPC-associated RanBP2/RanGAP1*SUMO1/Ubc9 complexes are distributed within the network of ER but not the tips of cell extensions. (A-D) HeLa cells were double stained with mAb414 and calreticulin antibody for labeling ER network (A), mAb414 and RanBP2 antibody (B), tubulin and calreticulin antibodies (C), and tubulin and RanBP2 antibodies (D) followed by immunofluorescence microscopy. The enlarged versions of inlets are shown at the bottom or top corner of each image (A-D). The arrows indicate the positions of the ALPC-associated RanBP2/RanGAP1*SUMO1/Ubc9 complexes that are most distant from the corresponding nucleus (D). The immunofluorescent images were taken using Olympus inverted IX81 widefield fluorescence microscope with U-Plan S-Apo 60×/1.35 NA oil immersion objective. Bar, 10 μm.

Previous studies using electron microscopy have shown that annulate lamellae are often continuous with ER network [1, 2, 4, 11, 12]. Furthermore, immunoelectron microscopy revealed that the mAb414-labeled cytoplasmic pore complexes are embedded in membranes [12, 60]. Moreover, double labeling immunofluorescence microscopy has shown that the cytoplasmic pore complexes stained by mAb414 are distributed within ER network labeled by the antibody against the ER membrane protein Sec61β, but not Golgi apparatus [12]. The localization of the RanBP2/RanGAP1*SUMO1/Ubc9 complexes at ALPCs prompted us to ask if these complexes are also distributed within the network of ER. To test this, HeLa cells were double stained with mAb414 and antibody specific to RanBP2 or calreticulin, a major calcium-binding protein primarily present in the ER lumen, and then analyzed by immunofluorescence microscopy. As expected, the cytoplasmic pore complexes labeled by mAb414 were almost completely overlapped with the staining of RanBP2, which was used a marker for the RanBP2/RanGAP1*SUMO1/Ubc9 complexes (Fig 4B). Consistent with the previous studies [12, 60, 62], we found that ~98% of the mAb414-labeled cytoplasmic foci of ALPCs are associated with the network of ER stained by anti-calreticulin antibody after analyzing 50 cells (~12 foci/cell) (Fig 4A).

It has been shown previously that RanBP2 is recruited to the edges of cell extensions by overexpression of the tumor suppressor protein APC (adenomatous polyposis coli) [63]. To test if the RanBP2/RanGAP1*SUMO1/Ubc9 complexes at ALPCs are also localized at the tips of cell extensions, HeLa cells were double stained with anti-tubulin antibody for labeling microtubules with a distribution throughout the cytoplasm and anti-calreticulin or anti-RanBP2 antibody followed by immunofluorescence microscopy. The ER network stained by anti-calreticulin antibody was broadly distributed in the cytoplasm but not obviously detected at the cell periphery including the edges of cell extensions (Fig 4C). Consistent with this, the cytoplasmic foci of ALPCs labeled by anti-RanBP2 antibody were absent at the tips of cell extensions after analysis of 50 cells (Fig 4D). Hence, we concluded that the RanBP2/RanGAP1*SUMO1/Ubc9 complexes at ALPCs are associated with the ER network but not the tips of cell extensions.

### SUMO1-modified RanGAP1 is equally distributed between nuclear and cytosolic fractions

**Fig 5:**
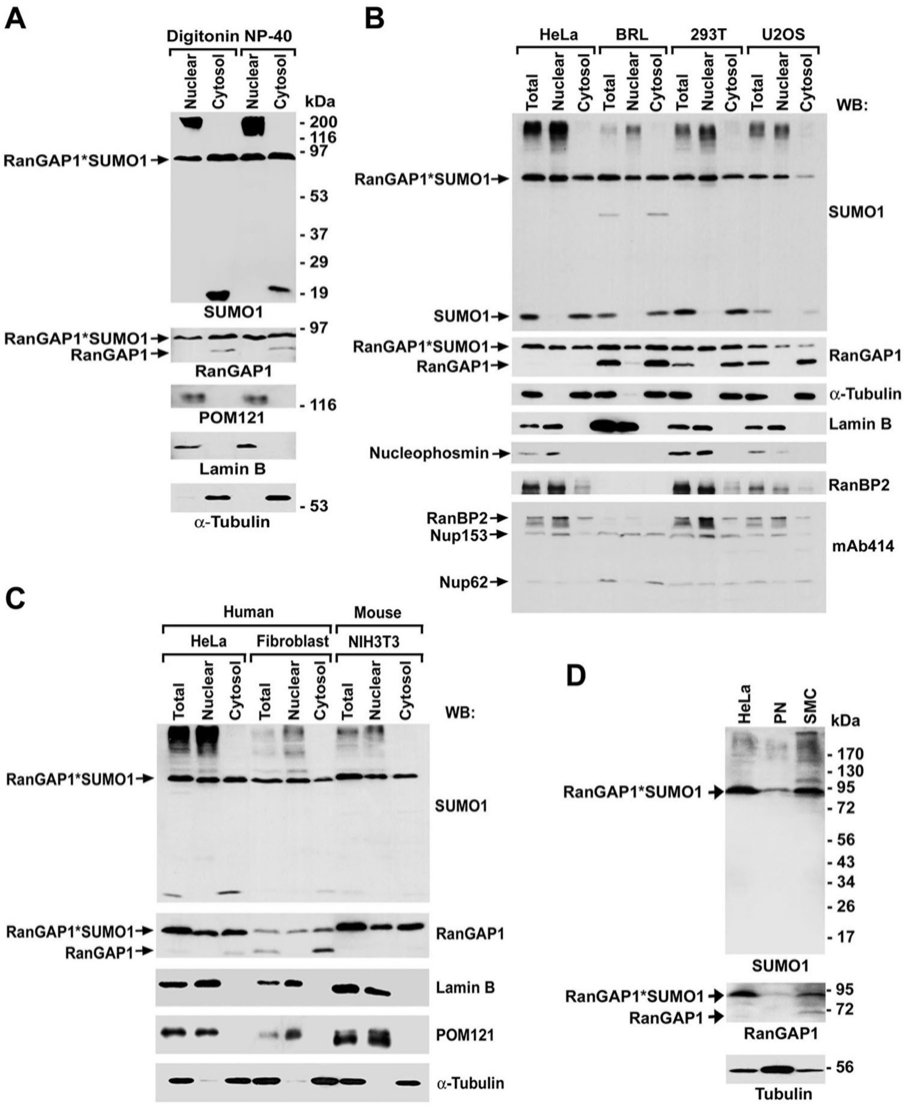
SUMO1-modified RanGAP1 is equally distributed between the nuclear and cytosolic fractions in a variety of mammalian cells. (A) HeLa cells were fractionated by two different methods using either digitonin or NP-40 as non-ionic detergent. The nuclear and cytosolic fractions were analyzed by immunoblotting with antibodies specific to SUMO-1, RanGAP1, α-tubulin as a marker for cytosolic proteins, lamin B as a marker for nuclear proteins, or POM121 as a marker for the NPC proteins. (B and C) Different types of tumor/cancer cells including HeLa, BRL, 293T and U2OS (B) and normal/non-tumorigenic fibroblasts including human GM03652 and mouse NIH3T3 (C) were fractionated by NP-40 method and then analyzed by immunoblotting with the indicated antibodies. (D) Total cell lysates of HeLa, PN and SMC cells were used for immunoblot analysis with the indicated antibodies.

To determine the distribution of the RanBP2/RanGAP1*SUMO1/Ubc9 complexes between the nuclear envelope and annulate lamellae, HeLa cells were fractionated into nuclear and cytosolic fractions using digitonin [35] or NP-40 method [58] followed by immunoblot analysis. We found that SUMO1-modified RanGAP1, a marker for the RanBP2/RanGAP1*SUMO1/Ubc9 complexes, was almost equally distributed between these two fractions (Fig 5A). This result was validated by immunoblot analysis of SUMO1-conjugates pulled down from both fractions (S2C Fig). To test if SUMO1-modified RanGAP1 is also evenly present between the two fractions in other types of cells, we fractionated four cancer cell lines (HeLa, BRL, 293T and U2OS) and two fibroblasts (GM03652 and NIH3T3) for immunoblot analysis. We found that SUMO1‐modified RanGAP1 was similarly distributed between nuclear and cytosolic fractions in five of the six different cells (including HeLa, BRL, 293T, human fibroblast, and NIH3T3), but not in U2OS cells, in which levels of SUMO1-modified RanGAP1 were obviously higher in nuclear fraction than in cytosolic fraction (Fig 5B and 5C). As expected, RanBP2, Nup153 and Nup62 were present in both fractions. Notably, SUMO1-modified RanGAP1 represented ~90% of RanGAP1 in HeLa, NIH3T3, PN and SMC cells but only ~50% in BRL, 293T, U2OS and GM03652 cells (Fig 5B–5D). To examine if there is any cross contamination between nuclear and cytosolic fractions, we analyzed the same fractions by immunoblotting with antibodies specific to Tubulin as a cytosolic marker, lamin B and nucleophosmin as nuclear markers, and POM121 as a marker for NPCs [7]. No cross-contamination was detected between nuclear and cytosolic fractions (Fig 5A–5C). Taken together with our immunofluorescence results (Figs 1 and 2), the presence of SUMO1-modified RanGAP1 and RanBP2 in both nuclear and cytosolic fractions provides the biochemical evidence that the RanBP2/RanGAP1*SUMO1/Ubc9 complexes are present at both NPCs and ALPCs.

### Upregulation of annulate lamellae causes a redistribution of both pore complexes and nuclear transport receptors from the nuclear envelope to annulate lamellae

**Fig 6:**
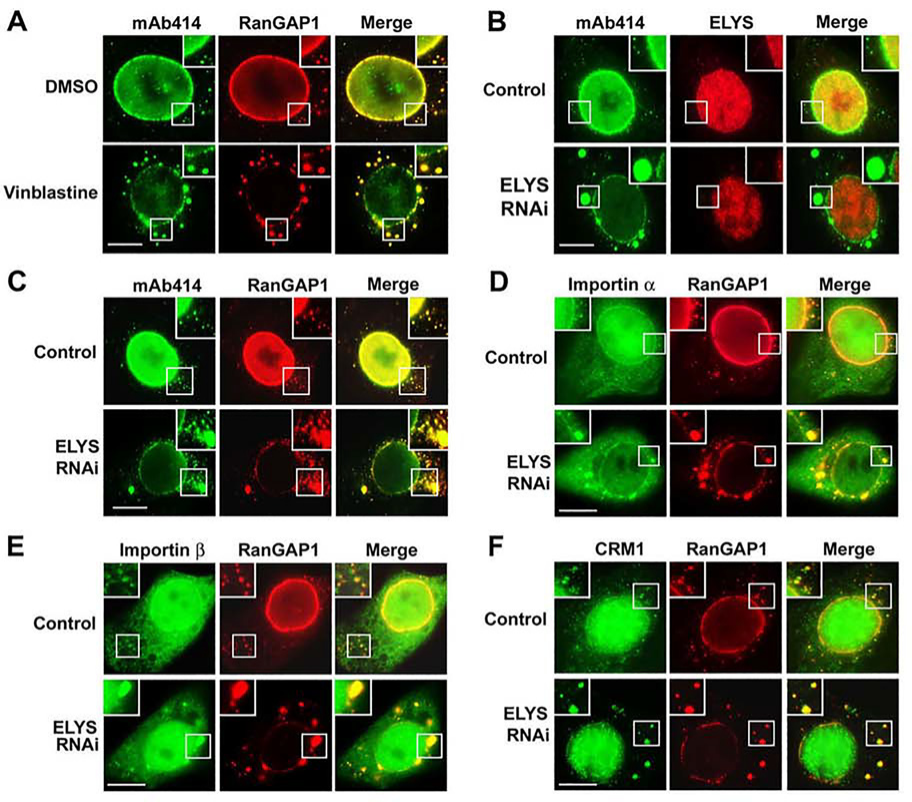
Upregulation of annulate lamellae causes a redistribution of both pore complexes and nuclear transport receptors from the nuclear envelope to annulate lamellae. (A) HeLa cells were treated with vinblastine or DMSO as a control and analyzed by immunofluorescence microscopy with mAb414 and anti-RanGAP1 antibodies. (B-F) HeLa cells were transfected with control or ELYS-specific siRNAs, double-labeled with mAb414 and anti-ELYS antibodies (B), mAb414 and anti-RanGAP1 antibodies (C), anti-importin α and anti-RanGAP1 antibodies (D), anti-importin β and anti-RanGAP1 antibodies (E), and anti-CRM1 and anti-RanGAP1 antibodies (F) followed by immunofluorescence microscopy. The boxes at the corner of each image represent the enlarged version of inlets. Bar, 10 μm.

Here we hypothesized that the distribution of the RanBP2/RanGAP1*SUMO1/Ubc9 complexes between NPCs and ALPCs is dependent on the relative levels of these two pore complexes. To test this hypothesis, we used three different approaches (ELYS RNAi, vinblastine treatment, and arginine deprivation) to upregulate annulate lamellae, which is known to increase the number of ALPCs but simultaneously decrease that of NPCs [1, 11, 52, 64–68]. Compared to control RNAi, ELYS RNAi [52] markedly decreased the staining of ELYS at NPCs as revealed by immunofluorescence microscopy (Fig 6B). Furthermore, our immunoblot analysis showed that levels of ELYS were dramatically knocked down in ELYS RNAi cells compared to control RNAi cells (S3 Fig). Consistent with our hypothesis, we showed that upregulation of annulate lamellae by the three different approaches not only caused a redistribution of pore complexes (labeled by mAb414) but also the RanBP2/RanGAP1*SUMO1/Ubc9 complexes (stained by RanGAP1 antibody) from the nuclear envelope to annulate lamellae (Fig 6A–6C and S4 Fig). Notably, previous studies using electron microscopy showed that the cytoplasmic pore complexes, which are accumulated or aggregated in cells treated with vinblastine sulfate, ELYS RNAi, or arginine deprivation (Fig 6A–6C and S4 Fig), are normally embedded within the ER-like membrane stacks and thus represent ALPCs rather than assembly intermediates [11, 52, 68].

Several karyopherins, including importin α/β and CRM1, have been shown previously to be associated with both NPCs and ALPCs in *Xenopus* eggs and mouse embryonic fibroblasts [60, 62, 69]. We therefore further hypothesized that the partition of importin α/β and CRM1 between NPCs and ALPCs is also determined by the relative levels of these two pore complexes. Consistent with this, our result revealed that ELYS RNAi decreased levels of importin α/β and CRM1 at NPCs but also increased their levels at ALPCs (Fig 6D–6F). Similarly, we showed that vinblastine treatment caused a redistribution of CRM1 from NPCs to ALPCs (S5 Fig). Hence, our results indicate that upregulation of annulate lamellae causes a redistribution of pore complexes including the RanBP2/RanGAP1*SUMO1/Ubc9 complexes and also nuclear transport receptors including importin α/β and CRM1 from the nuclear envelope to annulate lamellae.

### Upregulation of annulate lamellae with a decrease in NPCs and an increase in ALPCs reduces the rates of nuclear import and export

To test if upregulation of annulate lamellae affects nuclear transport, HeLa cells were first transfected with control or ELYS-specific siRNAs and then a construct encoding Rev-GR-GFP shuttling cargos [51]. Given the well-defined effect of ELYS RNAi on the assembly of both NPCs and ALPCs, we chose ELYS RNAi to upregulate annulate lamellae. Because the binding of ELYS to AT-rich chromatin sites represent the first step required for the post-mitotic assembly of NPCs, RNAi-knockdown of ELYS simultaneously causes a decrease in levels of NPCs and also an increase in levels of ALPCs [52, 64, 66, 70]. Additionally, RNAi-mediated knockdown of ELYS does not alter levels of nucleoporins, including Nup160, Nup358, Nup214, Nup153 and Nup133 [66]. As a widely used nuclear transport cargo reporter, Rev-GR-GFP contains an NES for CRM1-mediated export, a steroid-induced NLS for importin α/β-mediated import, and a GFP tag for monitoring its cellular distribution [51]. In the absence of steroid, CRM1-mediated export is dominant, leading to a primarily cytoplasmic distribution of Rev-GR-GFP. In the presence of steroid, importin α/β-mediated import becomes predominant, resulting in a nuclear accumulation of Rev-GR-GFP.

**Fig 7:**
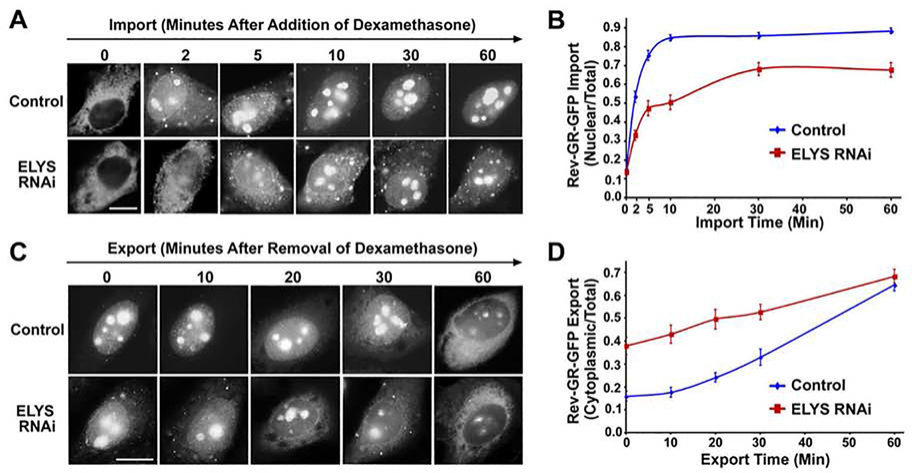
Upregulation of annulate lamellae by RNAi-knockdown of ELYS decreases the rates of both nuclear import and export. HeLa cells were transfected with control or ELYS-specific siRNAs and then with the construct encoding Rev-GR-GFP. (A and B) To evaluate the effect of ELYS RNAi on the rate of nuclear import, the transfected cells were treated with 0.25 μM of dexamethasone for the indicated times and analyzed by fluorescence microscopy (A). The histogram shows the nuclear to total signal ratios of Rev-GR-GFP (B). (C and D) To test if ELYS RNAi affects the rate of nuclear export, the transfected cells were treated with 0.25 μM of dexamethasone for 3 h to induce the nuclear accumulation of Rev-GR-GFP, washed with PBS, incubated with fresh medium for the indicated times, and analyzed by fluorescence microscopy (C). The histogram shows the cytoplasmic to total signal ratio of Rev-GR-GFP (D). Each bar indicates the mean value ± SEM (*N* = 60, Student’s *t* test) (B and D). Bar, 10 μm (A and C).

To exam if an increase in levels of annulate lamellae alters importin α/β-mediated import, the transfected cells were treated with 0.25 μM of dexamethasone for 0, 2, 5, 10, 30 and 60 min and then analyzed by fluorescence microscopy (Fig 7A and 7B). At 0 min, the nuclear to total signal ratio (N/T) of Rev-GR-GFP in control cells was similar to ELYS RNAi cells with a value of ~0.15. While the N/T ratio in control cells reached ~0.5 in <2 min, it took >10 min for the N/T ratio in ELYS RNAi cells to achieve this value. Further, the N/T ratio in control cells increased to its maximal value of ~0.85 in ~10 min, whereas the N/T ratio in ELYS RNAi cells reached its peak value of ~0.65 in ~30 min. Hence, the import rate in ELYS RNAi cells was about three times lower than in control cells.

To evaluate if induction of annulate lamellae changes CRM1-mediated export, the transfected cells were first incubated with 0.25 μM of dexamethasone for 3 h to achieve a primarily nuclear localization of Rev-GR-GFP. After removal of dexamethasone, the cells were incubated with fresh media to induce the export for 0, 10, 20, 30 and 60 min. At 0 min, the cytoplasmic to total signal ratio (C/T) of Rev-GR-GFP in control cells was ~0.15 compared to ~0.38 in ELYS RNAi cells (Fig 7C and 7D). This was consistent with the lower efficiency of import in ELYS RNAi cells compared to control cells (Fig 7A and 7B). At 60 min of export, the C/T ratio in control cells showed an increase of ~0.50, whereas that in ELYS RNAi cells displayed an increase of ~0.30, indicating that the rate of export in ELYS RNAi cells was markedly lower than in control cells. Altogether, upregulation of annulate lamellae and inhibition of NPC assembly by ELYS RNAi reduced the rates of both importin α/β-mediated nuclear import and CRM1-mediated nuclear export. This might be caused by a decrease in levels of NPCs, an increase in levels of ALPCs, or both.

### ALPCs serve as intermediate docking sites for importin α/β-mediated import complexes during nuclear import

The RanBP2/RanGAP1*SUMO1/Ubc9 complexes at NPCs mediate the hydrolysis of RanGTP within the importin β-RanGTP complexes docking on RanBP2, leading to the recycling of importin β and the subsequent assembly of the importin α/β-cargo import complexes at NPCs [44–46]. Given the localization of the RanBP2/RanGAP1*SUMO1/Ubc9 complex and importin α/β at both pore complexes (Figs 1–6), we hypothesized that the importin α/β-cargo import complex may also be assembled at and/or associated with ALPCs. However, we were unable to detect any obvious localization of Rev-GR-GFP cargos at both NPCs and ALPCs during dexamethasone-induced import in HeLa cells. This might be due to a transient association of the importin α/β-mediated import complexes with both pore complexes.

**Fig 8:**
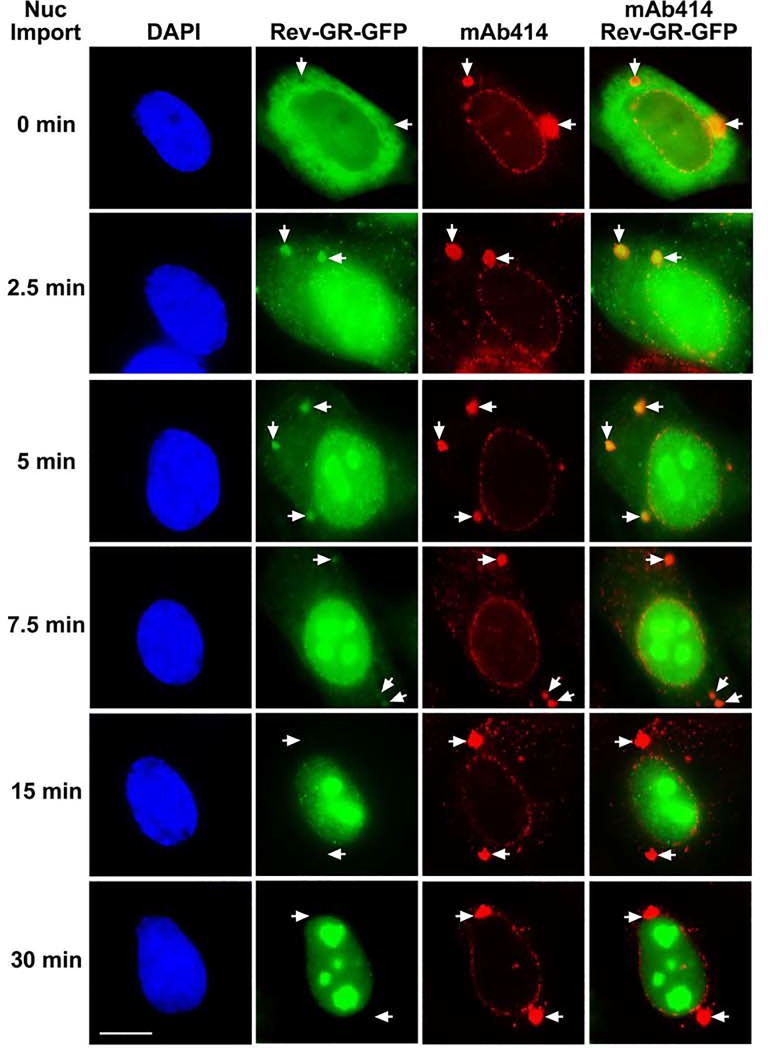
ALPCs function as intermediate docking sites for importin α/β-mediated import complexes during nuclear import. HeLa cells were transfected with siRNAs specific to ELYS to upregulate annulate lamellae and then with the construct encoding Rev-GR-GFP fusion proteins. The transfected cells were incubated with LMB for 2 h to inhibit CRM1-mediated export, treated with both dexamethasone (1 μM) and LMB to induce importin α/β-mediated import for the indicated times, and analyzed by immunofluorescence microscopy. At least 50 Rev-GR-GFP cells were analyzed for each time point of dexamethasone treatment to select o representative cell as shown in this figure. The arrows indicated the sites of ALPCs. Bar, 10 μm.

To test if we are able to detect the presence of Rev-GR-GFP cargos at ALPCs during steroid-induced nuclear import in cells with increased levels of ALPCs, we first transfected HeLa cells with ELYS-specific siRNAs to upregulate annulate lamellae and then the construct encoding Rev-GR-GFP shuttling cargos. To ensure that we monitor the Rev-GR-GFP cargos as import cargos rather than export cargos at ALPCs, we first treated the transfected cells with LMB for 2 h to block CRM1-mediated export [51] and then 1 μM dexamethasone to induce import for 0, 2.5, 5, 7.5, 15 and 30 min. We analyzed at least 50 Rev-GR-GFP cells by immunofluorescence microscopy for each time point and selected one representative cell as shown in Fig 8.

In the absence of steroid-induced nuclear import (0 min), we did not observe any obvious accumulation of Rev-GR-GFP at ALPCs labeled by mAb414 as the signals of Rev-GR-GFP within the foci of ALPCs were considerably weaker than the surrounding areas (Fig 8). At 2.5 and 5 min of import, a robust accumulation of Rev-GR-GFP at ALPCs was detected in nearly all the analyzed cells. The signal intensities of Rev-GR-GFP at ALPCs were markedly weaker at 7.5 min compared to those at 2.5 and 5 min and barely detectable at 15 and 30 min in almost all the transfected cells. Hence, our results suggest that ALPCs may act as intermediate docking sites for importin α/β-mediated import complexes that subsequently disassociate for nuclear import and accumulation through NPCs at least in cells with upregulation of annulate lamellae. As the association of import complexes with ALPCs was only transiently observed in ELYS RNAi cells with increased levels of ALPCs along with decreased levels of NPCs but not in control RNAi cells, one explanation is that the localization of import complexes at both NPCs and ALPCs might be too brief to be detected under normal conditions. At the same time, we cannot exclude the possibility that the importin α/β-cargo import complexes are also directly assembled at ALPCs.

### CRM1-mediated export complexes accumulate at both NPCs and ALPCs when the disassembly of these export complexes is inhibited by transient expression of RanQ69L GTPase mutant

**Fig 9:**
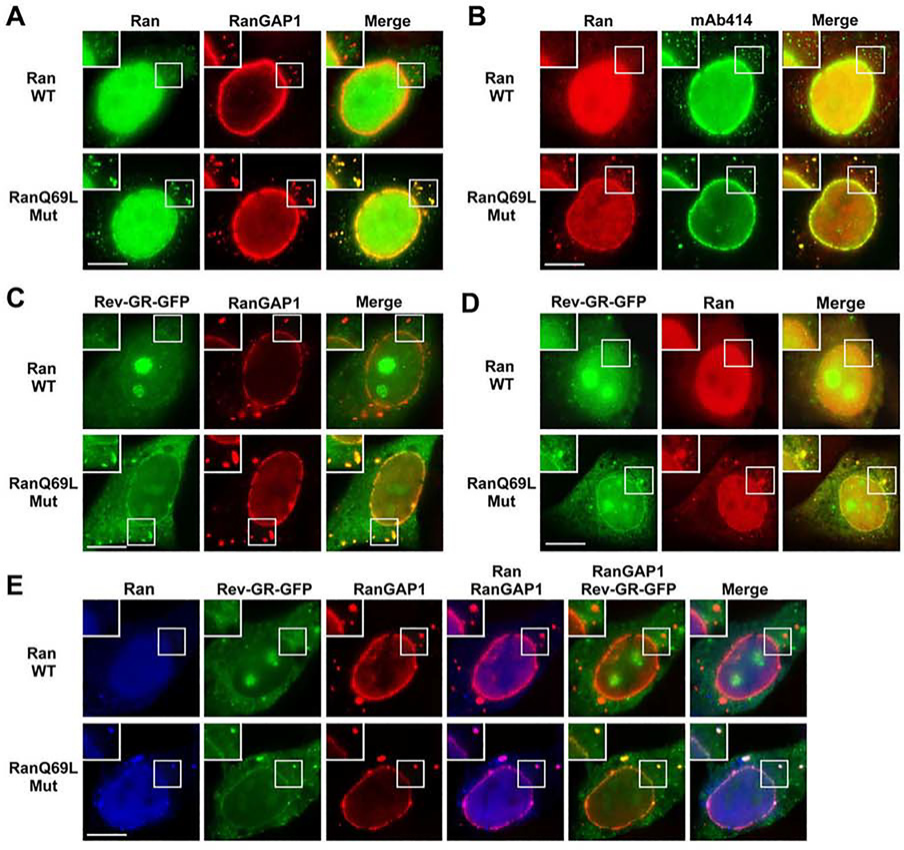
CRM1-mediated export complexes accumulate at ALPCs when the disassembly of these export complexes is inhibited by transient expression of RanQ69L mutant. (A and B) HeLa cells were transfected with the construct encoding FLAG-tagged Ran wild-type (WT) or RanQ69L GTPase mutant (Mut), double-labeled with anti-FLAG and RanGAP1 antibodies (A) or mAb414 (B) and analyzed by immunofluorescence microscopy. (C-E) HeLa cells were co-transfected with the constructs encoding Rev-GR-GFP and FLAG-tagged Ran WT or RanQ69L. The transfected cells were incubated with 1 μM dexamethasone to induce the nuclear accumulation of Rev-GR-GFP, washed with fresh medium to remove dexamethasone to initiate nuclear export, stained with anti-RanGAP1 antibodies (C), anti-FLAG antibodies (D), or both anti-RanGAP1 and anti-FLAG antibodies (E), and analyzed by immunofluorescence microscopy. Bar, 10 μm. The boxes at the top-left corner of each image reveal the enlarged version of inlets.

The CRM1-cargo-RanGTP export complexes are disassembled upon RanGTP hydrolysis mediated by the RanGAP1 and RanBP2 within the RanBP2/RanGAP1*SUMO1/Ubc9 complexes at NPCs and also by the RanGAP1 and RanBP1 in the cytosol [47, 71–75]. The localization of the RanBP2/RanGAP1*SUMO1/Ubc9 complexes and CRM1 at ALPCs let us hypothesize that CRM1-mediated export complexes may also be disassembled upon RanGTP hydrolysis at these cytoplasmic pore complexes. To test this, we first examined if ALPCs function in RanGTP hydrolysis. HeLa cells were transfected with the constructs encoding FLAG-tagged Ran WT and RanQ69L mutant, a GTPase mutant with a defect in catalyzing RanGTP hydrolysis and arrested in its GTP-bound form [32, 76]. Like other small GTPases, Ran cannot hydrolyze GTP to GDP at a physiologically significant rate by itself and requires interaction with RanGAP1 [32, 33, 76, 77]. We found that FLAG-Ran WT and FLAG-RanQ69L mutant were expressed at a level similar to endogenous Ran (S6 Fig). Our immunofluorescence microscopy showed that FLAG-RanQ69L mutant, but not FLAG-Ran WT, is localized at both NPCs and ALPCs labeled by anti-RanGAP1 antibody and mAb414 (Fig 9A and 9B). These results suggest that the hydrolysis of RanGTP at both NPCs and ALPCs causes the release of RanGDP from these pore complexes into the cytosol.

To test if the CRM1-cargo-RanGTP export complexes are disassembled upon RanGTP hydrolysis at ALPCs, HeLa cells were co-transfected with the constructs encoding the CRM1 cargos Rev-GR-GFP and FLAG-tagged Ran WT or RanQ69L mutant. The transfected cells were treated with dexamethasone to induce nuclear import of Rev-GR-GFP, washed with PBS to remove dexamethasone and to initiate export, and analyzed by immunofluorescence microscopy (Fig 9C–9E). We found that the Rev-GR-GFP export cargos were accumulated at both NPCs and ALPCs stained by anti-RanGAP1 antibody in the presence of FLAG-RanQ69L mutant but not FLAG-Ran WT, suggesting that inhibition of RanGTP hydrolysis prevents the disassembly of CRM1-cargo-RanGTP complexes at both pore complexes (Fig 9C). Furthermore, Rev-GR-GFP co-localized with FLAG-RanQ69L but not FLAG-Ran WT at NPCs and cytoplasmic foci (Fig 9D). To examine if these cytoplasmic foci represent ALPCs, the transfected cells were double-labeled with anti-FLAG and anti-RanGAP1 antibodies followed by immunofluorescence microscopy (Fig 9E). We found that Rev-GR-GFP cargos and FLAG-RanQ69L are co-localized at both ALPCs and NPCs labeled by anti-RanGAP1 antibody, whereas no accumulation of Rev-GR-GFP and FLAG-Ran WT was observed at both pore complexes. Because the RanQ69L mutant is arrested in its GTP-bound form and causes the disassembly of the importin α/β-cargo import complexes [29, 75], the Rev-GR-GFP cargos that co-localize with FLAG-RanQ69L at both NPCs and ALPCs likely represent the export cargos within the CRM1-cargo-RanGTP complexes other than the import cargo within the importin α/β-cargo complexes (Fig 9). Hence, our results suggest that the SUMO1-modified RanGAP1 and RanBP2 at both NPCs and ALPCs may function in disassembling the CRM1-cargo-RanGTP export complexes by coordinately activating the hydrolysis of RanGTP.

**Fig 10:**
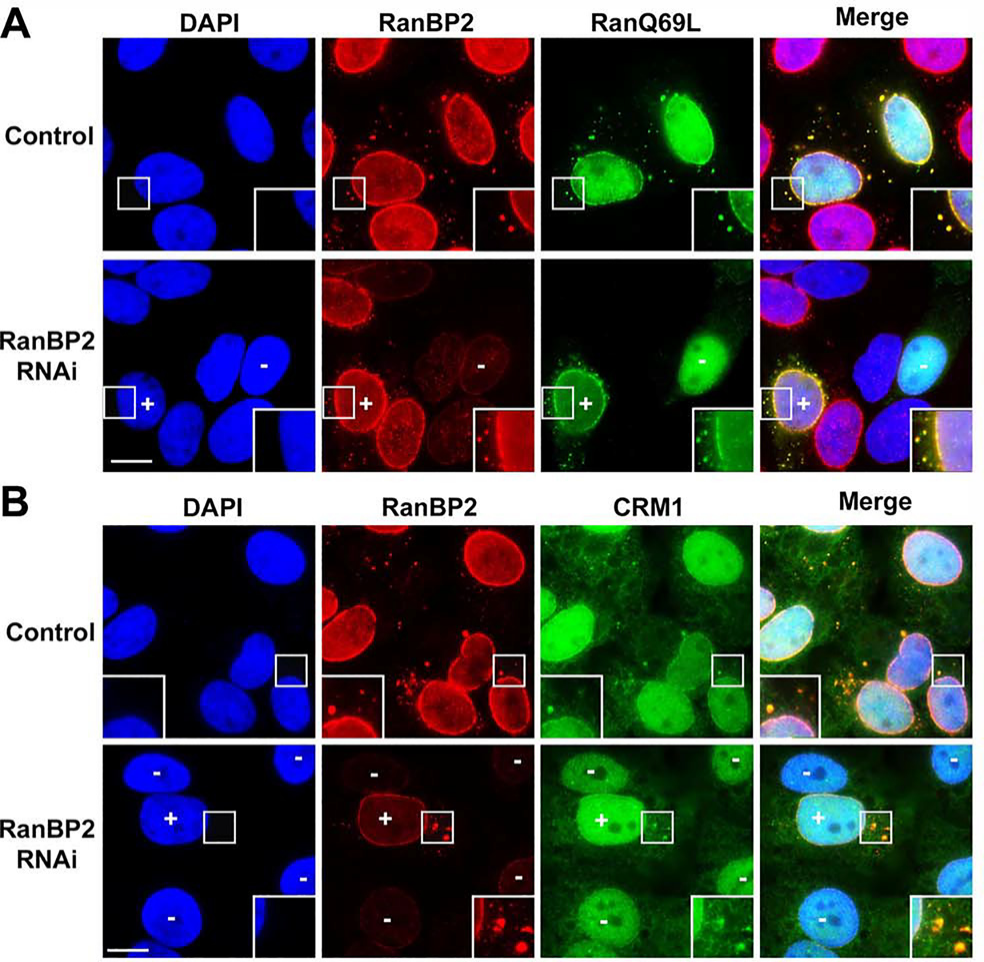
RanBP2 is required for the localization of RanGTP and CRM1 at NPCs and ALPCs. (A) HeLa cells were first transfected with control or RanBP2-specific siRNAs for 48 h and then the constructs encoding FLAG-tagged RanQ69L for 24 h followed by immunofluorescence microscopy with antibodies specific to RanBP2 and FLAG. (B) HeLa cells were transfected with control or RanBP2-specific siRNAs for 72 h and then analyzed by immunofluorescence microscopy with antibodies specific to RanBP2 and CRM1. In RanBP2 RNAi cells (lower panels), “-” indicates the cell with depletion of RanBP2, and “+” indicates the cell with levels of RanBP2 that are similar to those in control cells (upper panels). The boxes at the bottom corner of each image show the enlarged version of inlets. Bar, 10 μm.

To further elucidate the role of the RanBP2/RanGAP1*SUMO1/Ubc9 complexes at both NPCs and ALPCs in regulation of CRM1-mediated export complexes, we first used RNAi to knock down RanBP2 and then analyzed the localization of FLAG-tagged RanQ69L and endogenous CRM1 at these pore complexes by immunofluorescence microscopy (Fig 10). We found that RNAi-mediated depletion of RanBP2 almost completely abolished the localization of FLAG-RanQ69L and CRM1 at NPCs and ALPCs compared to control RNAi cells (Fig 10). This observation suggested that RanBP2, an essential component of the RanBP2/RanGAP1*SUMO1/Ubc9 complexes, may be required for the localization of RanGTP and CRM1 at both pore complexes. Hence, these results further support our model that the RanBP2/RanGAP1*SUMO1/Ubc9 complexes at both NPCs and ALPCs may play a pivotal role in the disassembly of CRM1-mediated export complexes by stimulating RanGTP hydrolysis.

## Discussion

**Fig 11:**
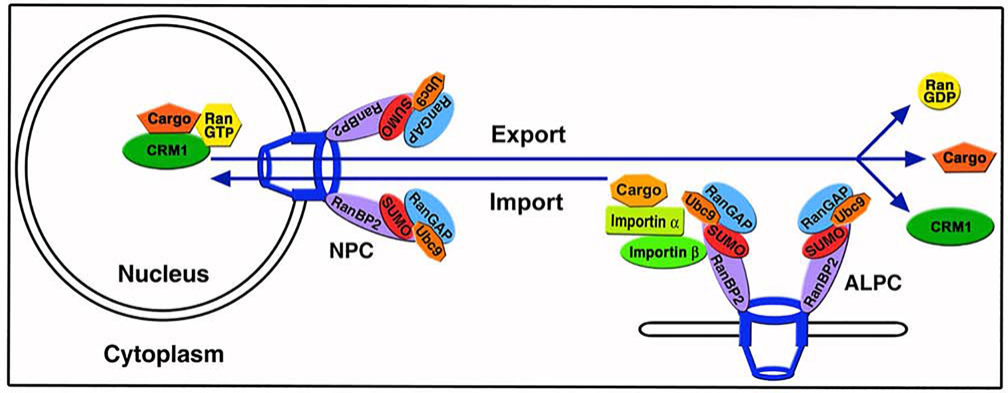
A model shows how ALPCs affects nuclear import and export in the cytoplasm. ALPCs may serve as the docking or assembling sites for importin α/β-mediated import complexes followed by their dissociation for nuclear import. On the other hand, the ALPC-associated RanBP2/RanGAP1*SUMO1/Ubc9 complexes may function in the disassembly of CRM1-mediated export complexes by mediating RanGTP hydrolysis.

In this study, we show that SUMOylation of RanGAP1 not only targets RanGAP1 to its known sites at NPCs but also to ALPCs by forming the RanBP2/RanGAP1*SUMO1/Ubc9 complexes in mammalian cells. We further demonstrate that upregulation of annulate lamellae is associated with a redistribution of pore complexes and nuclear transport receptors from the nuclear envelope to annulate lamellae and also decreases the rates of both nuclear import and export. Furthermore, ALPCs may serve as docking sites for importin α/β-mediated import complexes followed by their dissociation for nuclear import (Figs 8 and 11). Moreover, CRM1-mediated export complexes are also accumulated at ALPCs when the disassembly of these export complexes is inhibited by transient expression of the RanQ69L GTPase mutant, suggesting that RanGAP1/RanBP2-mediated RanGTP hydrolysis at ALPCs is required for the dissociation of the export complexes (Figs 9 and 11). Lastly, RanBP2 is required for the localization of RanGTP and CRM1 at ALPCs, supporting our model that the RanBP2/RanGAP1*SUMO1/Ubc9 complexes at ALPCs may play an essential role in regulation of CRM1-mediated export complexes (Figs 10 and 11). Therefore, our results provide a foundation for investigating how upregulation of annulate lamellae, which is characterized by a decrease in NPCs and an increase in ALPCs, reduces the rates of nuclear transport and also for elucidating the biological significance of the interaction between ALPCs and nuclear transport complexes in mammalian cells.

The reduced rates of both nuclear import and export in ELYS RNAi cells with upregulation of annulate lamellae (Fig 7) is highly likely caused by the decreased number of NPCs at the nuclear envelope. However, we cannot exclude the possibility that the increased number of ALPCs causes a temporary sequestration of both nuclear import and export complexes at these cytoplasmic pore complexes and therefore decreases the rates of nuclear import and export through NPCs. Therefore, upregulation of annulate lamellae in response to various treatments or conditions [1] may represent a novel mechanism to decrease the rates of nuclear transport. Conversely, it remains a possibility that ALPCs may also function as the cytoplasmic sites to facilitate a localized assembly of import complexes and also a site-specific disassembly of export complexes. This might be particularly important for cells with a relatively large cytoplasm such as nerve cells. Lastly, it might also be possible that upregulation of annulate lamellae in cells with RNAi-depletion of ELYS or under other conditions/treatments may cause a defect in NPC architecture. Further studies are required to elucidate how upregulation of annulate lamellae affects nuclear transport.

There is an intrinsic link between NPCs and ALPCs as both pore complexes are disassembled almost simultaneously into stable sub-complexes at the onset of mitosis and subsequently reassembled at the end of mitosis [12, 40, 78, 79]. In this study, we used three different approaches to increase levels of ALPCs along with a reduction of NPCs, including ELYS RNAi, vinblastine treatment and arginine deprivation. As ELYS is essential for the post-mitotic assembly of NPCs, RNAi-knockdown of ELYS greatly decreases levels of NPCs and therefore increases levels of ALPCs [52, 64, 66, 70]. In addition, a prolonged treatment of cells with vinblastine sulfate, an antitubulin drug that causes mitotic arrest by inhibiting microtubule polymerization [80], may increase levels of ALPCs through a mechanism similar to ELYS RNAi as microtubule polymerization is pivotal for the post-mitotic assembly of NPCs but not that of ALPCs [67]. On the other hand, little is known about how arginine deprivation affects the assembly of NPCs and ALPCs. Previous studies have shown that arginine deprivation causes cell-cycle arrest in S phase [81, 82]. Since NPC assembly also occurs during interphase [78], it is possible that arginine deprivation inhibits the assembly of NPCs in S phase and thus increases levels of ALPCs.

We notice that annulate lamellae are not only abundant in cells with high proliferative capacity [1, 12, 22] but also in cells under a permanent cell-cycle arrest such as murine brain-isolated neurons and heart-isolated cardiomyocytes [23, 24] (Fig 1D and 1E). In these cell-cycle arrested cells, annulate lamellae are highly unlikely to serve as a storage compartment of excess nucleoporins to support later cell proliferation. Therefore, it would be interesting to test whether high levels of ALPCs in these arrested cells causes a change in the rates of nuclear transport. Here we would like to emphasize that the major obstacle for functional analysis of ALPCs is the lack of approaches and/or tools to specifically inhibit or deplete these cytoplasmic pore complexes without affecting the nuclear pore complexes. This is because ALPCs do not contain any unique protein component when compared to NPCs. Hence, novel approaches and tools need to be developed in order to achieve a better understanding of annulate lamellae and their biological functions. We reason that the presence of annulate lamellae in nearly all types of cells and their upregulation upon various types of treatments and conditions provide a strong rationale to further investigate the biological functions of these mysterious organelles. These studies may help us gain novel mechanistic insights about both normal and abnormal cells that are associated with human diseases.

## Supporting Information

S1 Fig The structure of the vertebrate nuclear pore complex and its associated RanBP2/RanGAP1*SUMO1/Ubc9 complex. A) The diagram shows the vertebrate nuclear pore complex (NPC) that penetrates through the nuclear envelope at the place where outer nuclear membrane (ONM) and inner nuclear membrane (INM) fuse. The vertebrate NPC contains three transmembrane nucleoporins (orange) POM121, Ndc1 and gp210 that anchor the NPC at the nuclear envelope. The scaffold nucleoporins form the three ring-like structures including the cytoplasmic ring (red, the Nup107-160 complex), the central ring (green, the Nup93 complex), the nuclear ring (red, Nup107-160 complex). The central nucleoporins (green, the Nup62 complex) establish the permeability barrier. The peripheral nucleoporins (purple) form the cytoplasmic filaments and also the nuclear basket. The ALPC lacks three nucleoporins including ELYS, POM121 and Tpr that are highlighted by red arrows. B) The large nucleoporin RanBP2 (Nup358) forms the RanBP2/RanGAP1*SUMO1/Ubc9 complex with SUMO1-modified RanGAP1 and SUMO-conjugating enzyme Ubc9 at the NPC. RanBP2 contains a leucine-rich region (LRR) for its anchor to the NPC, four Ran-binding domains (RBD) for interaction with RanGTP, a zinc finger domain (ZnF), a kinesin-binding domain (KBD), several FG repeat motifs (dashes) for interaction with karyopherins, a cyclophilin A homologous domain (CyA), and an IR domain that contains the SUMO E3 ligase activity. The IR domain includes two internal repeats (IR1 and IR2). The RanGAP1 and RanBP2 within the RanBP2/RanGAP1*SUMO1/Ubc9 complex activates the hydrolysis of RanGTP to RanGDP, leading to the disassembly of the importin-RanGTP complex and the exportin-cargo-RanGTP complex.
(PDF)

S2 Fig SUMO1-modifed RanGAP1 localizes to both nuclear pore complexes and annulate lamellae pore complexes in HeLa cells. (A) HeLa cells stably expressing YFP-SUMO1 were analyzed by immunofluorescence microscopy using anti-RanGAP1 antibody or mAb414. The boxes at the top-right corner of each image show an enlarged version of inlets. Bar, 10 μm. (B) Immunoblot analysis of total cell lysates isolated from YFP-SUMO1 stable cells and control HeLa cells using antibodies specific to RanGAP1, SUMO1 and Tubulin. (C) The nuclear and cytosolic extracts of HeLa cells were used for immunoprecipitation with anti-SUMO1 mAb (21C7). The immunopurified SUMO1-conjugates were analyzed by immunoblotting with antibodies specific to RanGAP1 and SUMO1. The mouse ascites generated using SP2/0 myeloma cells were used for immunoprecipitation as control antibodies. Asterisk indicates the heavy or light chains of mAbs.
(PDF)

S3 Fig ELYS RNAi remarkably knocks down levels of ELYS. HeLa cells were transfected with either control or ELYS-specific siRNAs for 72 h followed by immunoblot analysis with anti-tubulin and anti-ELYS antibodies. The arrow indicates human ELYS with the expected size of ~250 kDa in control RNAi cells, whereas ELYS is greatly knocked down in ELYS RNAi cells. The asterisk indicates a non-specific protein band detected by anti-ELYS antibody.
(PDF)

S4 Fig Induction of annulate lamellae by arginine deprivation causes a redistribution of pore complexes from the nuclear envelope to annulate lamellae. HeLa cells were cultured in DMEM medium in the presence (control) or absence of arginine for 15 h, double labeled with anti-RanGAP1 antibody and mAb414, and then analyzed by immunofluorescence microcopy. Bar, 10 μm. The boxes at the top-left corner of each image show an enlarged version of inlets.
(PDF)

S5 Fig Upregulation of annulate lamellae by vinblastine treatment causes a redistribution of CRM1 from the nuclear envelope to annulate lamellae. HeLa cells were treated with vinblastine or DMSO as a control and analyzed by immunofluorescence microscopy. Bar, 10 μm. The boxes at the top-left corner of each image show an enlarged version of inlets.
(PDF)

S6 Fig Immunoblot analysis of FLAG-tagged Ran wild-type and RanQ69L mutant. HeLa cells were transiently transfected with the constructs encoding FLAG-tagged Ran wild-type (WT) or RanQ69L mutant, and analyzed by immunoblotting with antibodies specific to Ran, FLAG and Tubulin. Arrows indicate FLAG-Ran WT, FLAG-RanQ69L mutant and endogenous Ran.
(PDF)
