# Supplementary figures and images for: Molecular Characterization and Functional Analysis of Annulate Lamellae Pore Complexes in Nuclear Transport in Mammalian Cells
Source: PLoS One. 2015 Dec 7;10(12):e0144508. doi: 10.1371/journal.pone.0144508 (PMC4671610; doi:10.1371/journal.pone.0144508)

**S1 Fig.**

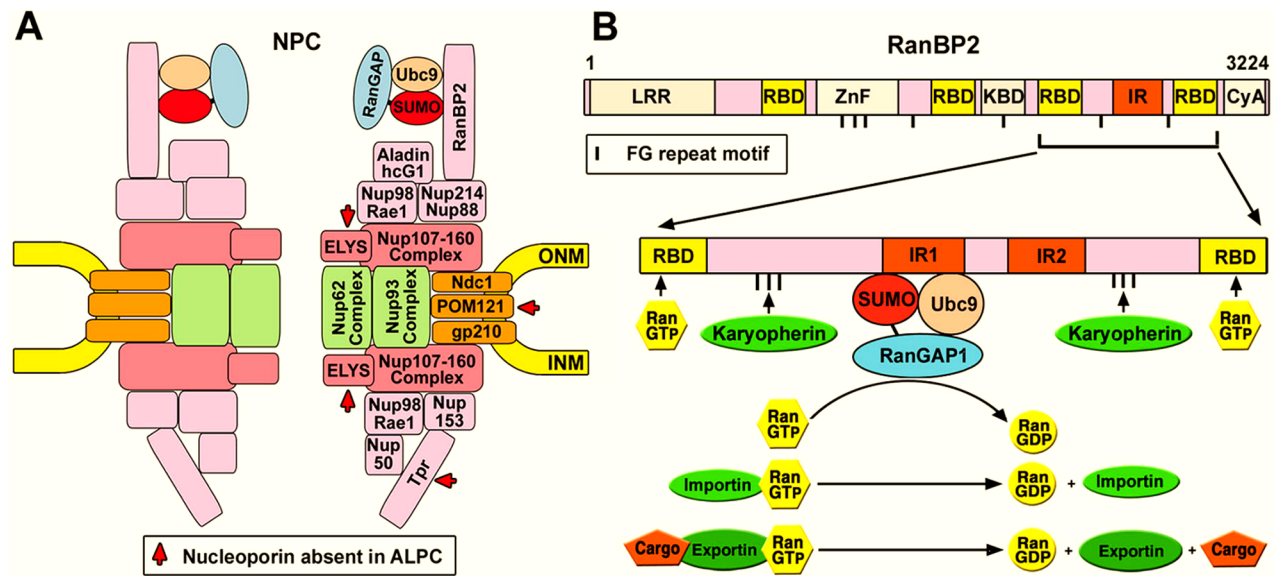

Supplement: S1 Fig — A) The diagram shows the vertebrate nuclear pore complex (NPC) that penetrates through the nuclear envelope at the place where outer nuclear membrane (ONM) and inner nuclear membrane (INM) fuse. The vertebrate NPC contains three transmembrane nucleoporins (orange) POM121, Ndc1 and gp210 that anchor the NPC at the nuclear envelope. The scaffold nucleoporins form the three ring-like structures including the cytoplasmic ring (red, the Nup107-160 complex), the central ring (green, the Nup93 complex), the nuclear ring (red, Nup107-160 complex). The central nucleoporins (green, the Nup62 complex) establish the permeability barrier. The peripheral nucleoporins (purple) form the cytoplasmic filaments and also the nuclear basket. The ALPC lacks three nucleoporins including ELYS, POM121 and Tpr that are highlighted by red arrows. B) The large nucleoporin RanBP2 (Nup358) forms the RanBP2/RanGAP1*SUMO1/Ubc9 complex with SUMO1-modified RanGAP1 and SUMO-conjugating enzyme Ubc9 at the NPC. RanBP2 contains a leucine-rich region (LRR) for its anchor to the NPC, four Ran-binding domains (RBD) for interaction with RanGTP, a zinc finger domain (ZnF), a kinesin-binding domain (KBD), several FG repeat motifs (dashes) for interaction with karyopherins, a cyclophilin A homologous domain (CyA), and an IR domain that contains the SUMO E3 ligase activity. The IR domain includes two internal repeats (IR1 and IR2). The RanGAP1 and RanBP2 within the RanBP2/RanGAP1*SUMO1/Ubc9 complex activates the hydrolysis of RanGTP to RanGDP, leading to the disassembly of the importin-RanGTP complex and the exportin-cargo-RanGTP complex. (PDF) [file pone.0144508.s001.pdf]

S2 Fig.

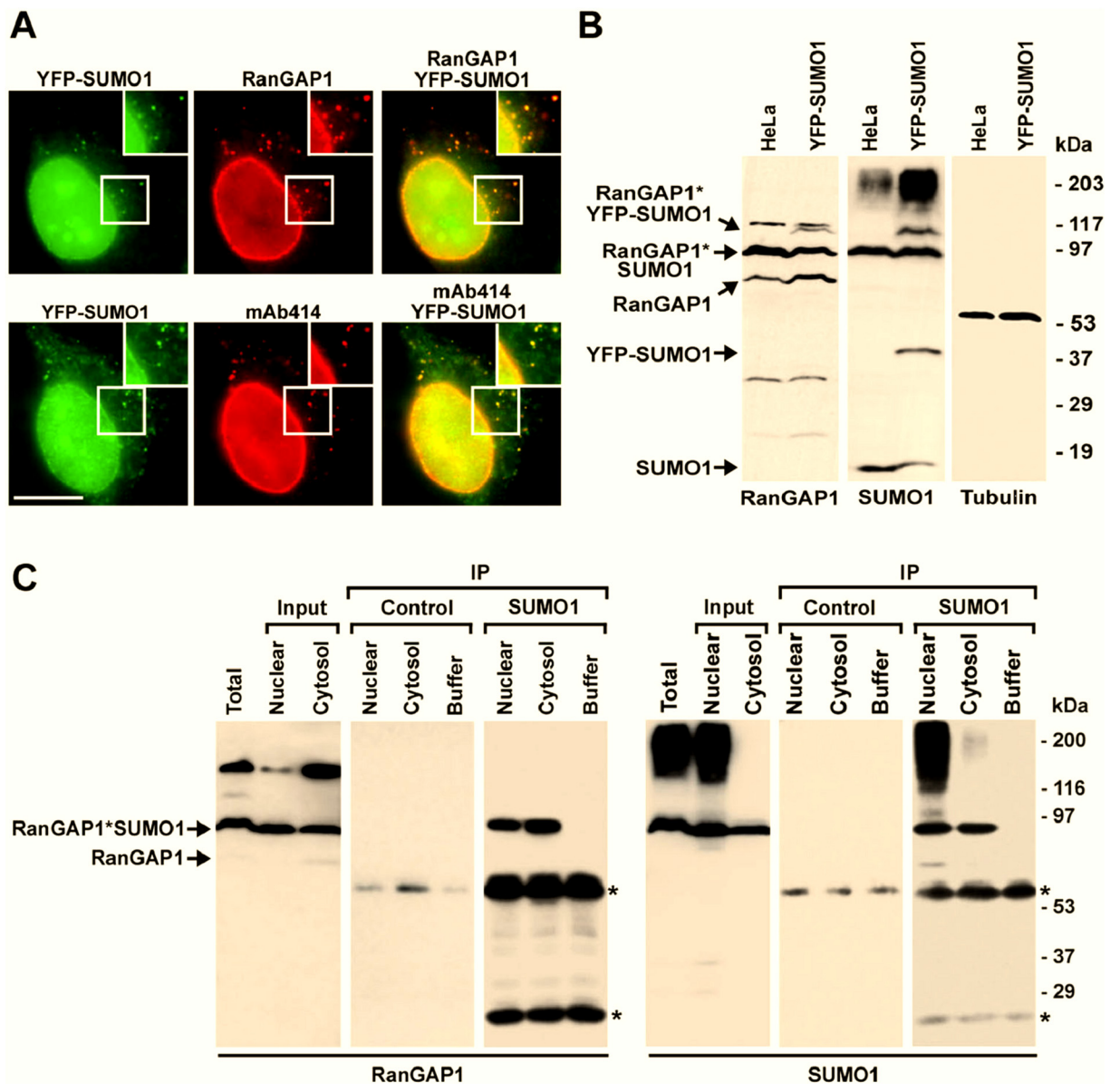

Supplement: S2 Fig — (A) HeLa cells stably expressing YFP-SUMO1 were analyzed by immunofluorescence microscopy using anti-RanGAP1 antibody or mAb414. The boxes at the top-right corner of each image show an enlarged version of inlets. Bar, 10 μm. (B) Immunoblot analysis of total cell lysates isolated from YFP-SUMO1 stable cells and control HeLa cells using antibodies specific to RanGAP1, SUMO1 and Tubulin. (C) The nuclear and cytosolic extracts of HeLa cells were used for immunoprecipitation with anti-SUMO1 mAb (21C7). The immunopurified SUMO1-conjugates were analyzed by immunoblotting with antibodies specific to RanGAP1 and SUMO1. The mouse ascites generated using SP2/0 myeloma cells were used for immunoprecipitation as control antibodies. Asterisk indicates the heavy or light chains of mAbs. (PDF) [file pone.0144508.s002.pdf]

**S3 Fig.**

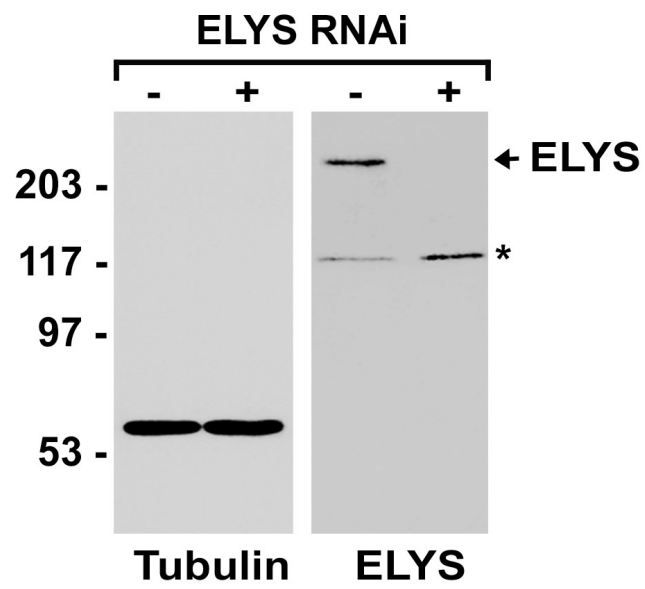

Supplement: S3 Fig — HeLa cells were transfected with either control or ELYS-specific siRNAs for 72 h followed by immunoblot analysis with anti-tubulin and anti-ELYS antibodies. The arrow indicates human ELYS with the expected size of ~250 kDa in control RNAi cells, whereas ELYS is greatly knocked down in ELYS RNAi cells. The asterisk indicates a non-specific protein band detected by anti-ELYS antibody. (PDF) [file pone.0144508.s003.pdf]

**S4 Fig.**

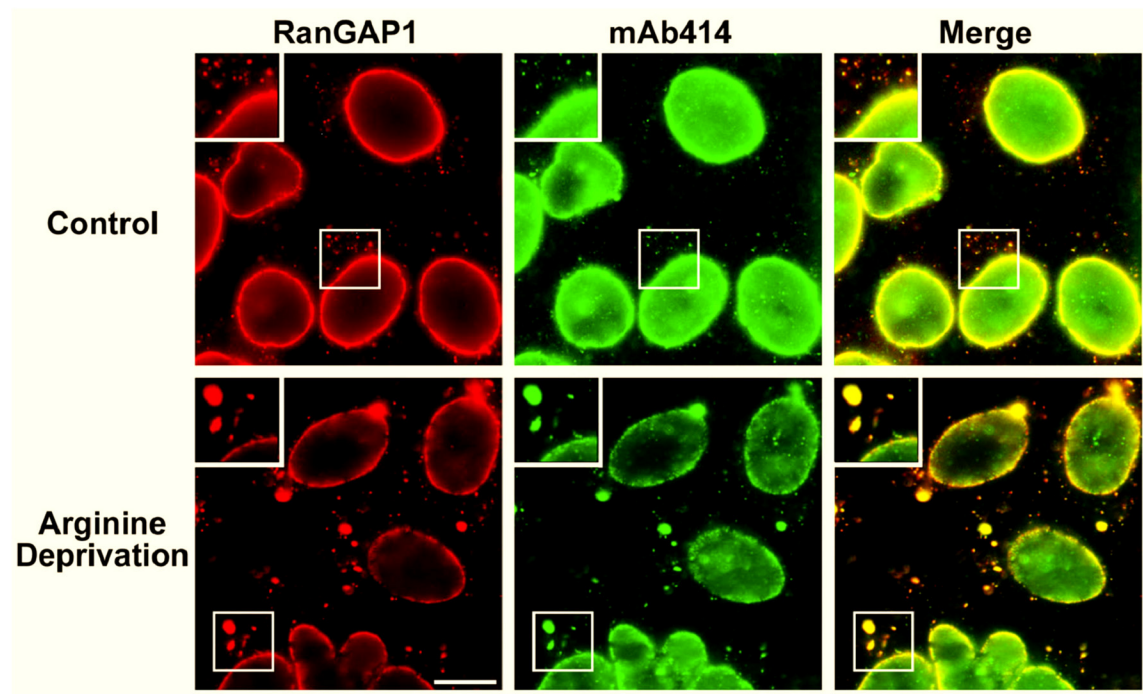

Supplement: S4 Fig — HeLa cells were cultured in DMEM medium in the presence (control) or absence of arginine for 15 h, double labeled with anti-RanGAP1 antibody and mAb414, and then analyzed by immunofluorescence microcopy. Bar, 10 μm. The boxes at the top-left corner of each image show an enlarged version of inlets. (PDF) [file pone.0144508.s004.pdf]

**S5 Fig.**

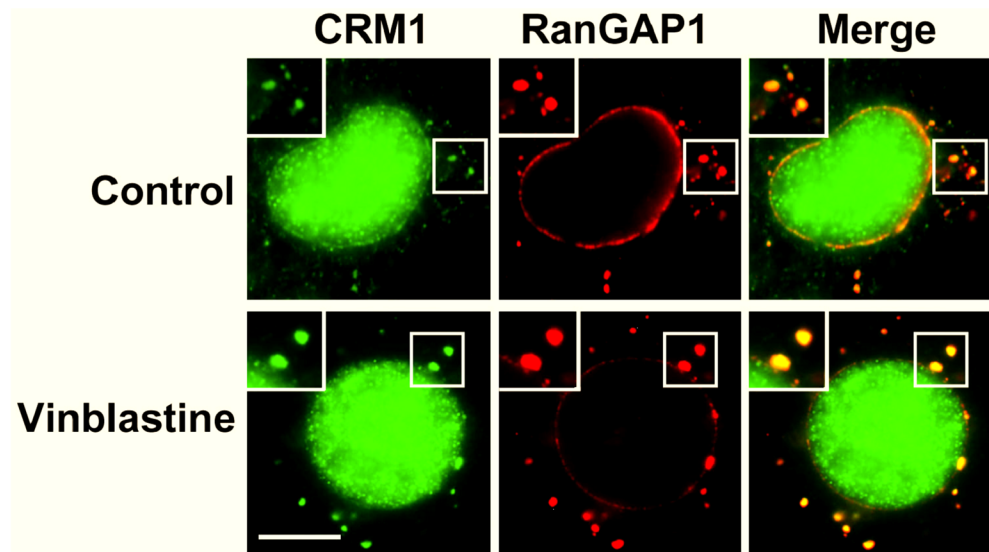

Supplement: S5 Fig — HeLa cells were treated with vinblastine or DMSO as a control and analyzed by immunofluorescence microscopy. Bar, 10 μm. The boxes at the top-left corner of each image show an enlarged version of inlets. (PDF) [file pone.0144508.s005.pdf]

**S6 Fig.**

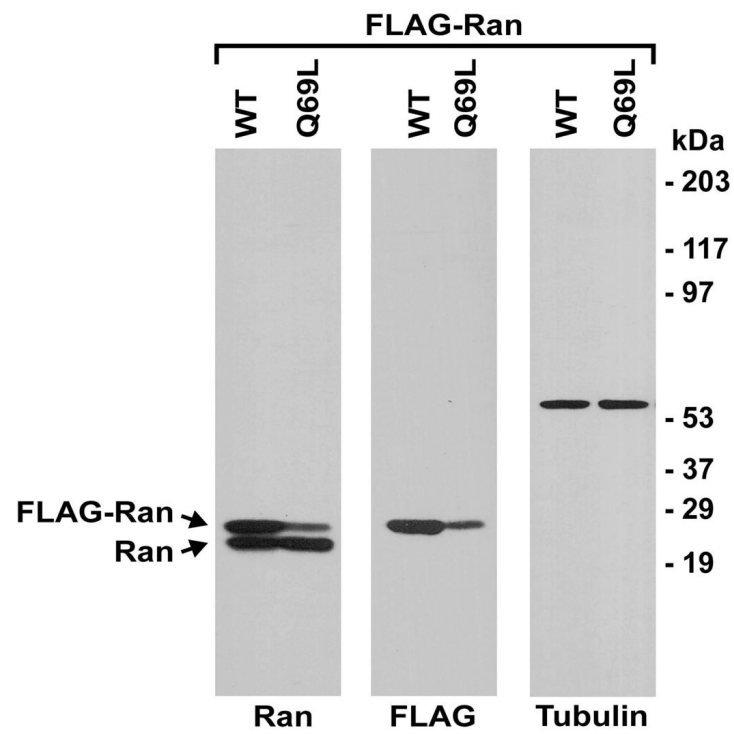

Supplement: S6 Fig — HeLa cells were transiently transfected with the constructs encoding FLAG-tagged Ran wild-type (WT) or RanQ69L mutant, and analyzed by immunoblotting with antibodies specific to Ran, FLAG and Tubulin. Arrows indicate FLAG-Ran WT, FLAG-RanQ69L mutant and endogenous Ran. (PDF) [file pone.0144508.s006.pdf]
